# The S100A8/A9–NETosis feedback loop in sepsis: potential mechanisms, immune crosstalk, and therapeutic targeting

**DOI:** 10.3389/fimmu.2026.1790788

**Published:** 2026-04-17

**Authors:** Sini Chen, Jingying Lin, Jiahao Huang, Liehua Deng, Yuanli Zhang

**Affiliations:** Department of Critical Care Medicine, Affiliated Hospital of Guangdong Medical University, Zhanjiang, China

**Keywords:** sepsis, S100A8/A9, neutrophil extracellular trap, immune crosstalk, therapeutic strategy

## Abstract

Sepsis has a high incidence and mortality rate, bringing about a high global burden. Recent research has demonstrated that the S100A8/A9–NETosis feedback loop is considered a central regulator of sepsis. However, few studies have integrated these elements into a unified ‘S100A8/A9–NETosis feedback loop’. Meanwhile, a systematic description of this loop in the interaction between myeloid cells and platelets, as well as organ damage, has not been established. This review systematically proposes the S100A8/A9–NETosis positive feedback loop as a core pathological mechanism and explores its potential pathways in sepsis. Specifically, we examine how this loop drives neutrophil-mediated hyperinflammation, monocyte/macrophage polarization, immune paralysis (mediated by dendritic cells and myeloid-derived suppressor cells), and immune thrombosis (triggered by platelets). We also evaluate how this loop contributes to organ-specific injury and discuss potential therapeutic strategies, providing new perspectives for sepsis treatment.

## 1 Introduction

Sepsis is a leading cause of death among critically ill patients (1, 2). This means that the host’s reaction to the infection becomes uncontrolled, leading to multiple organ dysfunction. Even with the ongoing developments in antimicrobial treatment and supportive care, the in-hospital mortality rate remains excessively high (3–5). This is largely attributed to the complex and conflicting immune dysregulation. The pathogenesis involves a gradual shift from a hyperinflammatory state to immune paralysis, culminating in the development of secondary infections and organ dysfunction (6). Damage-associated molecular patterns (DAMPs) play a central role in the progression of systemic inflammation and endothelial damage (7, 8).

The S100A8/A9 heterodimer, a danger-associated molecular pattern (DAMP), is crucial in the development of sepsis (9). It is primarily stored in myeloid cells, including neutrophils and monocytes, and is rapidly released on cell activation or necrosis (10, 11). High levels of S100A8/A9 are significantly associated with disease severity, such as organ failure and mortality (12). In addition, it promotes neutrophil recruitment and contributes to the formation of neutrophil extracellular traps (NETs) (13). NETs are complex structures of DNA, histones, neutrophil elastase (NE), and S100A8/A9, and have become a fundamental part of sepsis pathogenesis (14). This process of NET formation, is referred to as NETosis, a form of programmed cell death specific to neutrophils (15). Although it is a significant part of natural antibacterial resistance, its exacerbation or persistent presence may contribute to immunosuppression, endothelial cell damage, and tissue injury (16).

Despite the extensive description of S100A8/A9 and NETosis as independent triggers of sepsis, a critical knowledge gap is how these two interact to drive the transition from a localized immune response to a self-sustaining cascade. In 2007, Vogl et al. showed that S100A8/A9 is an endogenous ligand of TLR4 and directly participates in the occurrence of sepsis (17). Urban et al. initially proved that S100A8/A9 is an important component protein of NETs, providing physical evidence for subsequent studies (18). Tang et al. demonstrated through inhibitors, gene knockout, and other methods that S100A8/A9 induces platelet pyroptosis and subsequent NET formation in sepsis, proposing the core concept of the S100A8/A9–NETosis positive feedback loop (12). Therefore, S100A8/A9 can induce NETosis, and NETosis, in turn, promotes the release of S100A8/A9, forming a positive feedback loop of inflammation.

This review offers a novel conceptual framework by proposing an integrated S100A8/A9–NETosis feedback loop that may amplify sterile and infectious inflammation. Unlike previous reviews that focus on either molecule in isolation, we specifically address: (1) how S100A8/A9 promotes NETosis through multiple mechanisms and how NETs in turn release S100A8/A9 to perpetuate the loop; (2) how this loop mediates the crosstalk between myeloid cells and platelets and drives organ damage; and (3) the therapeutic significance as well as clinical translation challenges of targeting this loop. This integrated perspective provides a mechanistic basis for understanding sepsis as a self-sustaining inflammatory loop, identifying novel intervention points.

## 2 Mechanistic insights into the S100A8/A9–NETosis loop

### 2.1 Molecular basis of the S100A8/A9–NETosis loop

S100A8/A9 is one of the main triggers of NETosis in the sepsis microenvironment (13). Various endogenous and exogenous danger signals regulate the expression and release of S100A8/A9, including DAMPs and pathogen-associated molecular patterns (PAMPs) (11). Neutrophils are stimulated by classic inflammatory stimuli, such as bacterial lipopolysaccharide (LPS), tumor necrosis factor-α (TNF-α), high mobility group box-1 (HMGB1), S100A8/A9, and so on. S100A8/A9 is regarded as an amplifier of inflammatory signaling, rather than a major initiator (11, 19). It involves a positive feedback mechanism in which S100A8/A9 interacts with pattern recognition receptors (PRRs) to trigger NETosis (20).

**Figure 1:**
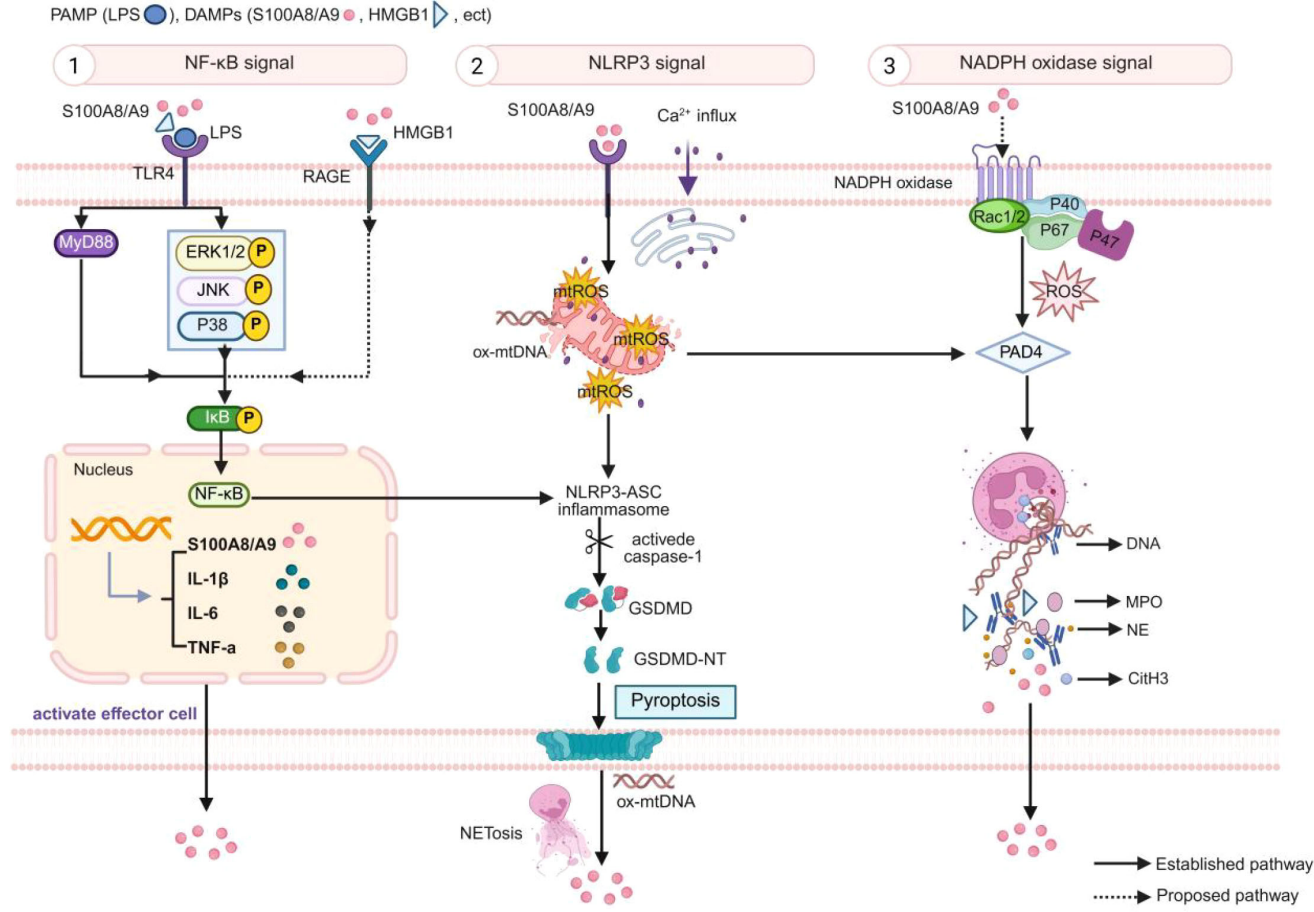
Molecular basis of the S100A8/A9–NETosis loop. (1) NF-κB signaling: PAMPs or DAMPs primarily bind to TLR4–MyD88 and TLR4–MAPK cascades (ERK1/2, JNK, and p38). DAMPs also trigger the RAGE–MAPK pathway. These signals promote the translocation of NF-κB (p65/p50) to the nucleus and release S100A8/A9, IL-1β, IL-6, and TNF-α. (2) NLRP3 signaling: S100A8/A9–TLR4 increases Ca^2+^ influx and mtROS accumulation. NF-κB and mtROS trigger the NLRP3–GSDMD signaling cascade, inducing pyroptosis. In addition, mtROS activates PAD4 signaling to induce NETosis, while ox-mtDNA triggers extracellular NETosis, both inducing the release of S100A8/A9. (3) NOX signaling: S100A8/A9 is proposed to promote NOX complex assembly and increase ROS production, which in turn activates PAD4 to induce NETosis and release NET-associated proteins. The released S100A8/A9, acting as a secondary DAMP, reactivates the above pathways, forming a positive feedback loop that perpetuates the inflammatory response. Created using BioRender.

As shown in **Figure 1**, activation of S100A8/A9 commonly begins with the combination of TLR4 and RAGE (21). This binding activates MyD88-dependent and MAPK/ERK signaling cascades, which stimulates the transcription of NF-κB (P50/P65) (22). This pathway not only increases the expression of pro-inflammatory cytokines, including TNF-α and IL-1β, but also induces S100A8/A9 expression (23, 24). The TLR4–NF-κB pathway acts as a central priming hub, lowering the activation threshold for NETosis while simultaneously upregulating NLRP3 and pro-IL-1β to facilitate pyroptosis in sepsis-induced acute kidney injury (AKI) (25). According to the existing evidence, the S100A8/A9–TLR4 pathway has been demonstrated in both *in vitro* and *in vivo* conditions when sepsis occurs. In contrast, the direct role of the S100A8/A9–RAGE pathway in sepsis remains less clear. For instance, Wang et al. demonstrated that extracellular vesicle (EV)-bound S100A8/A9 from septic shock patients could induce acute lung injury (26). While S100A8/A9 stimulation in RAGE-knockout mice significantly reduced levels of TNF-α and IL-6, it failed to reduce neutrophil infiltration. These findings suggest that S100A8/A9 may only partially signal through the RAGE pathway, particularly concerning cytokine regulation rather than cellular recruitment.

Disruption of Ca²^+^ homeostasis and mitochondrial dysfunction are also the essential steps of the inflammatory response mediated by S100A8/A9 (27). TLR4 activation leads to the Ca²^+^ release from the endoplasmic reticulum and the formation of Ca^2+^ influx (28). This leads to the sustained activation of protein kinase C (PKC), which promotes the citrullination of histone H3 (CitH3) by peptidylarginine deiminase 4 (PAD4) (29). Furthermore, this Ca²^+^ influx triggers mitochondrial reactive oxygen species (mtROS) release and induces mitochondrial dysfunction (30). The accumulated mtROS can activate PAD4-mediated signaling, triggering the release of proteins into the extracellular space (31). Moreover, mtROS also can activate the NLRP3–GSDMD signaling pathway to trigger cell pyroptosis and NETosis, while also leading to the release of oxidized mitochondrial DNA (ox-mtDNA) (32). When ox-mtDNA is released into the extracellular space, it can trigger neutrophil activation and NETosis (12). Besides, S100A9 also triggers ERK1/2-Drp1 signaling downstream of the TLR4/RAGE signaling pathway, leading to mitochondrial respiratory dysfunction (33). These interconnected cellular processes (NETosis, pyroptosis, and oxidative damage) deepen our understanding of sepsis-induced immune dysregulation.

S100A8/A9 can also induce NETosis via the NADPH oxidase (NOX)-dependent pathway (34). The research by Berthier et al. demonstrated that S100A8 directly binds to the cytochrome b558 component of the NADPH oxidase (NOX2) complex in PMA-stimulated PLB985 cells (35). This provides a molecular basis for S100A8/A9-mediated NOX activation. In addition, S100A8/A9 may interact with NOX subunits (p67phox, p47phox, and Rac2), thereby enhancing cytosolic ROS production and driving PAD4-mediated chromatin disassembly and NETosis (36). During NETosis, large amounts of S100A8/A9 are released into the extracellular microenvironment. Evidence from animal models supports this. Pharmacological inhibition of S100A8/A9 in a mouse model of myocardial infarction (MI) can suppress NOX and reduce ROS generation, thereby inhibiting NETosis and limiting myocardial damage (37). Although this model is based on myocardial infarction, these data suggest that the S100A8/A9–NOX–ROS–NETosis loop is a conserved inflammatory pathway. Pharmacological inhibition of NOX and ROS also suppresses S100A8/A9 downstream effects, indicating a feedback loop between S100A8/A9 and NOX (38). However, whether the S100A8/A9–NOX pathway operates similarly in septic conditions remains to be experimentally confirmed.

All these mechanisms show that the S100A8/A9–NETosis feedback loop is a vital part of the control of inflammatory imbalance. This also triggered our exploration of this loop in myeloid cells and platelets.

### 2.2 Immune cell crosstalk in the S100A8/A9–NETosis loop

#### 2.2.1 Neutrophils: initial activation and inflammatory response

**Figure 2:**
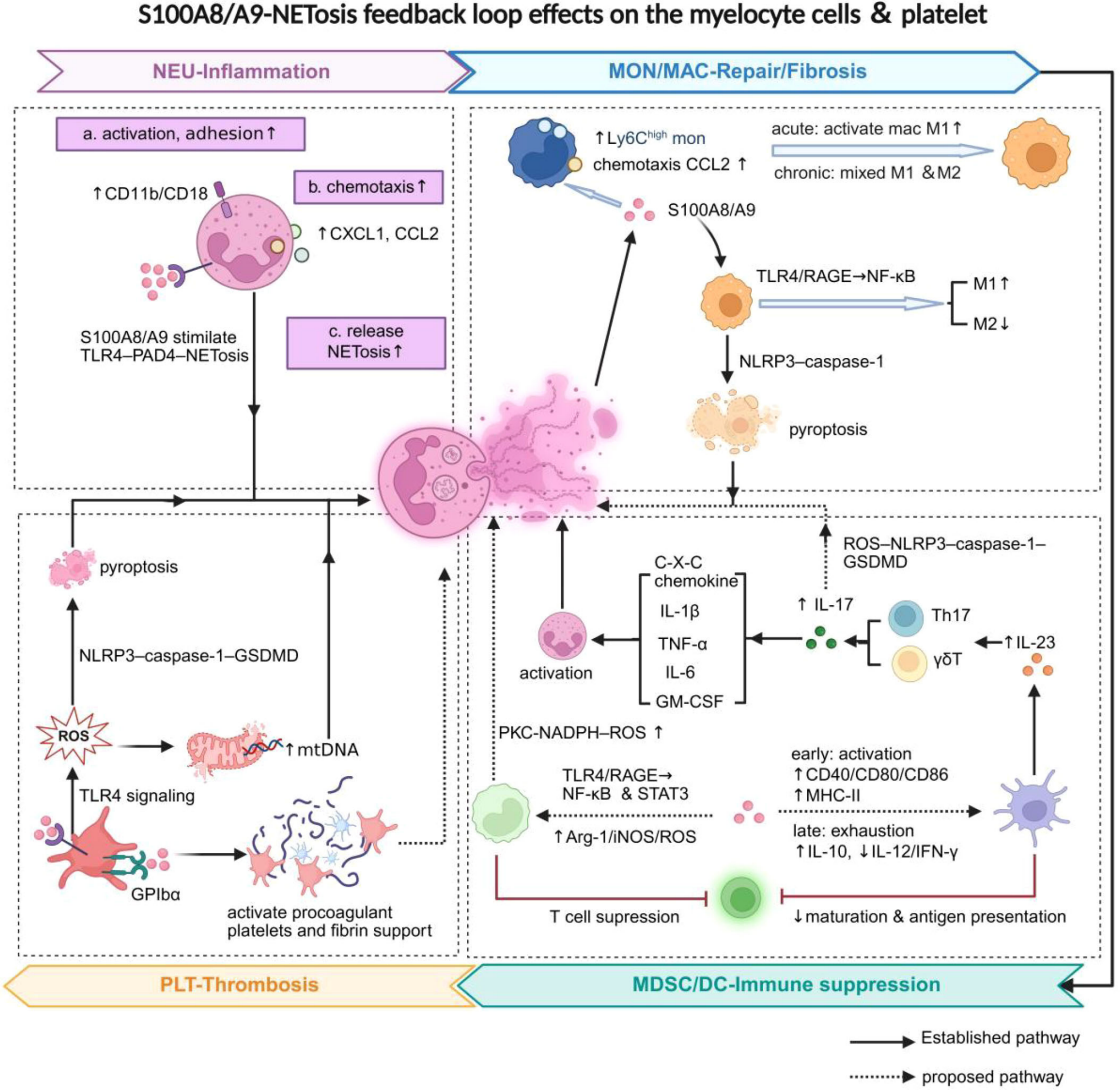
Regulation of immune crosstalk in the S100A8/A9–NETosis loop. (1) NEU-mediated inflammation: S100A8/A9 activates neutrophils, increasing adhesion (CD11b/CD18), chemotaxis (CXCL1 and CXCL2), and NETosis through TLR4–PAD4 signaling. (2) MON/MAC-mediated repair and fibrosis: S100A8/A9 recruits Ly6C^high^ monocytes, can promote macrophage polarization via NF-κB signaling, and can also induce NETosis via the NLRP3-mediated pyroptosis pathway. (3) MDSC/DC-mediated immunosuppression: S100A8/A9 may activate DCs, enhancing CD40, CD80, CD86, and MHC-I expression, which promotes IL-23–IL-17 signaling and NETosis early on. Later, DCs become exhausted (↑IL-10, ↓IL-12, and IFN-γ), suppressing T-cell function. S100A8/A9 may also activate MDSCs (↑Arg-1, iNOS, and ROS) through NF-κB or STAT3 signaling, amplifying immunosuppression. (4) PLT-mediated thrombosis: S100A8/A9 activates TLR4–NLRP3 signaling, triggering pyroptosis and NETosis, and may also bind to GPIbα to promote procoagulant platelet and NETosis. Created using BioRender.

Neutrophils are the major source of S100A8/A9, accounting for 30-40% of soluble cytosolic proteins (13). Under septic conditions, this relationship evolves into a pathogenic feed-forward circuit that sustains the inflammatory response. As shown in **Figure 2**, exogenous S100A8/A9 can stimulate neutrophil adhesion, activate β_2_ integrins (CD11b/CD18), and chemokines CXCL1 and CXCL2 to promote migration (39, 40). For instance, monosodium urate monohydrate crystals, a potent trigger of gout, can induce a significant release of S100A8/A9. This release subsequently enhances the activation of β_2_ integrin and chemokine production, thereby promoting the recruitment of neutrophils to the inflammatory tissue (40). The experimental data on inhibiting S100A9 support this view. This inhibition significantly reduced the production of CXC chemokines in circulation and lung tissue, and alleviated the pathological features of sepsis-related lung injury (41). Such chemokine-driven recruitment is a dual effect: while intended for pathogen clearance, the resulting platelet-leukocyte complex aggregation often precipitates septic cardiomyopathy and lethal tissue injury (42).

This mechanistic framework is very consistent with the preclinical studies. For example, S100A8/A9 inhibition significantly reduced the generation of CXC chemokines in the peripheral and lung tissues, thereby mitigating the pathological aspects of sepsis-related lung injury (41). In addition, S100A8/A9 is an endogenous ligand, mainly activating the TLR4–PAD4 signaling pathway in neutrophils (43, 44). This interaction lowers the activation threshold for NETosis, thereby promoting the degradation of neutrophil chromatin and the release of intracellular proteins. The intracellular released proteins include S100A8/A9, which will once more stimulate the neighboring cells to initiate an inflammatory response. Moreover, through PAD4 knockout and disruption of the S100A8/A9–TLR4 signaling pathway, it has been shown that it may substantially reduce ventilator-induced lung injury (VILI) and improve survival in animal models (43, 45). The clinical applicability is also evident. Plasma S100A8/A9 concentrations in patients with severe sepsis or septic shock are strongly correlated with NETosis markers (MPO-DNA and dsDNA), which indicates the clinical relevance of the pathway (12).

By enhancing neutrophil recruitment, promoting NETosis via the TLR4–PAD4 pathway, and triggering immunothrombosis, S100A8/A9 serves as a crucial mediator in the pathogenesis of sepsis. This loop represents an early amplification mechanism of the septic inflammatory response, making it a promising target for early-stage therapeutic intervention.

#### 2.2.2 Monocytes/macrophages: context-dependent polarization and tissue repair

When monocytes and macrophages are stimulated with pro-inflammatory factors such as TNF-α, IL-1β, IL-6, and CCL2, the expression and release of S100A8/A9 will increase significantly (46). However, unlike the rapid response of neutrophils, the monocyte-macrophage lineage exhibits significant plasticity. They can dynamically switch between pro-inflammatory and immune repair in response to changes in the inflammatory microenvironment. During this process, S100A8/A9 becomes a key factor in regulating the recruitment of monocytes and macrophages.

Multiple disease models have confirmed that elevated levels of S100A8/A9 are closely associated with the recruitment of inflammatory monocytes. For example, the single-cell transcriptomic analysis of atrial tissue from patients with atrial fibrillation (AF) revealed this result. Research has shown that the highly expressed S100A8/A9 was associated with the accumulation of Ly6C^high^ monocytes, a process mainly mediated by the TLR4–NF-κB signaling pathway (47). Its chemotactic activity was also confirmed in a mouse model of osteoarthritis (OA): intra-articular injection of S100A8 could promote the recruitment of Ly6C^high^ monocytes and upregulate CCL2, while knocking out the S100A9 gene significantly weakened monocyte migration (48). During the subacute phase of sepsis, single-cell sequencing analysis also showed that the percentage of S100A9^+^ monocytes peaked 72 hours after CLP surgery, suggesting significant enrichment of these cells at later stages (49). Moreover, extracellular vesicles rich in S100A9 can activate the RAGE signaling within macrophages, induce the expression of M1-type markers such as iNOS and CD86 in sepsis-induced ALI, thereby exacerbating local inflammatory responses (26). These results support the notion that S100A8/A9 is a potent chemotactic molecule and plays a specific role in the development of the mononuclear phagocyte phenotype.

In acute sepsis, S100A8/A9 predominantly induces M1 polarization. This leads to a hyperproinflammatory response, which is intended to enhance the antibacterial response and cytokine release (50). Nevertheless, its immunoregulatory function is highly context-dependent. Under specific cytokine conditions (M-CSF stimulation), in contrast, S100A9 promotes the expression of M2-associated transcriptional programs, facilitating tissue reconstruction and fibrosis (51). Another pattern can be seen in the model of metabolic inflammation. S100A9 induced M1 polarization in mice on a high-fat diet through its promotion of the TLR4–NF-κB signaling pathway and suppression of M2 polarization (52). The situation is even more complicated. S100A9 causes macrophages to become pro-inflammatory or mixed activation state via the TLR4–NLRP3 signaling cascade and also induces pyroptosis (53). The process of macrophage pyroptosis is accompanied by NETosis, both contributing to the release of large amounts of S100A8/A9 and ox‑mtDNA (54). These S100A8/A9 and ox-mtDNA, in turn, activate TLR4 and RAGE on neighboring immune cells, thus forming a positive feedback loop of inflammation. This sustained activation prevents the usual resolution of inflammation, favoring the M1-mediated antibacterial reaction at the expense of widespread organ injury (55).

Overall, this has shown that S100A8/A9 plays a dual role in the biological functions of monocytes/macrophages. In acute inflammation, it primarily stimulates TLR4–NF-κB and RAGE–NF-κB signaling pathways, which provide M1 polarization and exacerbate the inflammation. However, during the chronic stage or at the resolution stage, it can be inclined toward M2 polarization, which helps to repair tissue and fibrosis.

#### 2.2.3 Dendritic cells: transition from activation to tolerogenic exhaustion

Dendritic cells (DCs) are at the center of the S100A8/A9–NETosis loop. They play an essential role in the connection between innate sensing and adaptive immune responses in sepsis (56). In the acute phase, DCs may detect extracellular S100A8/A9 via the TLR4 and/or RAGE pathways, which subsequently causes a complex and dose- and time-dependent immune response (57). Low and moderate concentrations of S100A8/A9 may induce DC activation and differentiation. It is defined by co-stimulatory molecules (CD80, CD86, CD40) and antigen-presenting molecule (MHC-I) upregulation, thereby increasing the antigen-presenting ability (56, 57).

As a result, activated DCs increase the release of interleukin-23 (IL-23), further stimulating the growth and activation of Th17 and γδT cells, eventually causing interleukin-17 (IL-17) to rise dramatically (58). The IL-17 signal enhances the inflammatory microenvironment by inducing the expression of C-X-C chemokines (including CXCL1, CXCL2, CXCL4, CXCL5, CXCL8) and pro-inflammatory cytokines (including IL-1β, TNF-α, IL-6, and GM-CSF), thereby facilitating neutrophil recruitment and activation (58). This regulatory framework is supported by studies in the models of inflammatory diseases. The Sjögren’s dry eye disease (SjDED) model has shown that S100A8/A9 or STAT3 signaling can be pharmacologically inhibited, suppressing DC activation and IL-23 production, demonstrating the plasticity of DCs in response to the S100A8/A9 signal (59). It is also important to note that the role of IL-17 is not only specific to adaptive immunity. Research has shown that IL-17 can reduce the oxidative stress and inflammasome activation of myeloid cells via the ROS–NLRP3 pathway in colorectal cancer cells, which could augment local ROS burden and may reduce the inflammatory threshold that promotes NETosis (60). This mechanism has not been fully explained in septic DCs, but it is a valid point of connection for the DC-driven IL-17 signaling, which likely promotes the activation of neutrophils and NETosis in the S100A8/A9–NETosis loop indirectly.

A functional shift in DCs toward a tolerant and exhaustion-like phenotype accompanies the progressive accumulation of extracellular S100A8/A9 in sepsis. The increased secretion of IL-10 and the decreased secretion of IL-12 and interferon-γ (IFN-γ) are the typical features of this phenotypic change (61, 62). This functional and metabolic exhaustion seriously impairs T-cell activation, rendering the host susceptible to secondary infections (63). Notably, such a functional change in DCs is linked to the impaired clearance of NETosis. Due to the increase in S100A8/A9, reprogramming of DCs causes a decline in DNASE1L3 secretion, consequently resulting in failure of extracellular DNA degradation (64). The persistence of cfDNA further enhances the S100A8/A9–NETosis feedback loop, thus hampering the resolution of inflammation and leading to immune dysregulation.

The plasticity of DC provides a strategic window for intervention. By restoring DC-mediated NET clearance and re-establishing the Th1/Th17 balance, targeted therapy may prevent the transition from acute sepsis to chronic immunosuppression in immune homeostasis.

#### 2.2.4 Myeloid-derived suppressor cells: driving immune suppression

Myeloid-derived suppressor cells (MDSCs) are a heterogeneous population of immature myeloid cells that increase markedly in malignancy, chronic inflammation, and the late immunosuppressive phase of sepsis, where they exert potent inhibitory effects on both innate and adaptive immune responses (65, 66). In sepsis, the proportion of Gr1^+^CD11b^+^ MDSCs increases significantly during the immunosuppressive stage, and clinical as well as experimental studies indicate a close association between elevated S100A9 expression and MDSC accumulation (66). Notably, extracellular S100A8/A9 function as key autocrine and paracrine regulators of MDSC differentiation, survival, and functional maturation (65).

Mechanistically, extracellular S100A8/A9 can bind to TLR4 or RAGE, activating downstream NF-κB and MAPK signaling to stimulate MDSC differentiation and survival while simultaneously suppressing T cell activation (67–69). Models of colorectal cancer (CRC) have demonstrated that heightened S100A9 expression associates with MDSC expansion to enhance immunosuppression by pathways of both RAGE–MAPK and TLR4–NF-κB (69). Notably, targeting the receptor seems to be disease and situation-specific. Deletion of RAGE did not affect S100A8-induced differentiation or suppressive ability of MDSCs in a model of collagen-induced arthritis (CIA), but TLR4 deficiency completely abolished these effects, indicating that TLR4 plays an effective role in this inflammation (70). Furthermore, temporal regulation enhances the effect of S100A8/A9 on the biology of MDSC. *In vitro* experiments have revealed that the incubation of bone marrow progenitors with S100A8 required 6 days to trigger the differentiation of bone marrow progenitors into MDSCs. However, the incubation of MDSCs in LPS took 6 to 8 days, activating MDSCs to suppress T cells (70). The present findings suggest a cooperative role between S100A8/A9 and microbial signals in the propagation as well as immunosuppressive programming of MDSCs in hyperinflammatory conditions like sepsis.

At the molecular scale, S100A8/A9 maintains the immunosuppressive phenotype of MDSCs by upregulating arginase-1 (Arg-1), inducible nitric oxide synthase (iNOS), and ROS with the help of STAT3 activation and redox-sensitive signaling (70). Meanwhile, S100A8/A9 promotes phosphorylation of p38 MAPK and PKC that may facilitate NOX assembly and continuous ROS production, thus magnifying MDSC-mediated immune suppressions (66). With NET formation being highly dependent on the strictly maintained intracellular Ca^2+^ influx and ROS presence, the overproduction of ROS and depletion of arginine induced by MDSCs may disrupt NETosis, impair pathogen elimination, and contribute to immune homeostasis issues in sepsis (65, 66). The key point is that the connection between S100A8/A9-driven MDSCs and NETosis in inducing immune paralysis is still unclear. There are three possibilities worth further investigation: (1) S100A8/A9 stimulates MDSC-derived ROS to trigger NET formation and induce paralysis; (2) NETs enhance the inflammatory response, aggravating immune paralysis; or (3) there is mutual reinforcement between the two pathways.

Clinical observations suggest the pathological relevance of this loop. In patients with late-stage sepsis, plasma and intracellular S100A9 are positively correlated with the expansion of peripheral MDSCs, which reaches its peak around 24 hours following the onset of the disease (64). Pharmacological modulation of redox balance or arginine metabolism, using ROS scavengers such as N-acetylcysteine (NAC) or arginase inhibitors like nor-NOHA, has been linked to elevated levels of cfDNA, as well as PAD4^+^ cells, which are in line with a partial recovery of NETosis capability (66, 71). However, ROS are derived from multiple sources, and clearing the ROS does not necessarily attenuate the immunosuppression driven by MDSCs. In addition, the release of the S100A8/A9 by MDSC can induce the differentiation of macrophages and DCs via TLR7 signaling during both the initial and late stages of the disease, further inhibiting immune response (72). But the interaction between S100A8/A9 and MDSCs with other cells remains unknown in sepsis.

#### 2.2.5 Platelets: linking inflammation to immunothrombosis

Platelets, which are traditionally known to be involved in hemostasis, have become an important effector in the immune dysregulation observed in sepsis (73). S100A8/A9 is a potent alarmin and a marker of sepsis, initiating platelet pyroptosis (74). S100A8/A9 induces platelet pyroptosis mediated via the TLR4–ROS–NLRP3–caspase-1 signaling pathway in mice with CLP-induced sepsis, which is followed by mitochondrial membrane rupture and oxidative stress (12). The ox-mtDNA resulting from this mechanism further promotes NETosis and facilitates the formation of pro-inflammatory and pro-coagulant states (12, 75). Paquinimod inhibits S100A8/A9–TLR4 signaling, significantly decreasing platelet-induced pyroptosis and immunothrombosis in mice with sepsis, and significantly enhancing survival conditions (12). Furthermore, S100A8/A9 binds the platelet receptor glycoprotein Ib alpha chain (GPIbα), facilitating the development of platelet procoagulants, amplifying fibrin deposition and platelet thrombosis, which is mediated by the immune system (76). In mouse bacterial endotoxin experiments, blocking platelet GPIbα reduces neutrophil activation, chemotaxis, and inflammatory infiltration, suggesting that inhibiting GPIbα may also affect NET release (77). All these data indicate that platelet pyroptosis contributes to exacerbating S100A8/A9–NETosis-mediated thromboinflammatory responses in sepsis.

The S100A8/A9-NETosis feedback loop, from the hyper-inflammatory state of neutrophils, the promotion of cell polarization and repair by monocytes/macrophages, the immunosuppression driven by DCs/MDSCs and the promotion of thrombosis by platelets. This illustrates that the feedback loop is neither a single linear process nor the effect of a single cell, but rather a process in which multiple cells influence each other, driving multi-organ dysfunction in sepsis.

### 2.3 Organ-specific pathophysiology of the S100A8/A9–NETosis loop in sepsis

#### 2.3.1 Early phase: pulmonary injury and endothelial dysfunction

The S100A8/A9–NETosis loop plays a key role in organ-specific damage in sepsis. While it acts systemically, its effects are highly organ-specific, influenced by the unique microvascular structure and cellular dynamics of the lungs, liver, kidneys, heart, and brain. This loop starts with acute endothelial disruption and develops to chronic organ dysfunction.

The initial organ that becomes involved in the S100A8/A9–NETosis loop of sepsis is the lung (78). When neutrophils are activated, the first capillary bed encountered by neutrophils is the pulmonary vasculature, which becomes the primary site of injury (79). S100A8/A9 plays a role in facilitating acute respiratory distress syndrome (ARDS) in LPS-induced acute lung injury (ALI) by mediating TLR4 and RAGE signaling (78, 80). Moreover, the markers of pulmonary vascular endothelial integrity, including occludin and VE-cadherin, are decreased in sepsis through the inhibition of the P38/STAT3/ERK signaling pathways, and restored through S100A9 deficiency, suggesting that the endothelial barrier impairment is also connected to S100A8/A9 expression (81). This leads to the subsequent release of NETs into the microcirculation, releasing CitH3 and NE, further increasing vascular permeability and aggravating alveolar epithelial damage (80). Preclinical evidence suggests that blockade of TLR4 or neutralization of S100A8/A9 can limit protein-rich pulmonary edema and enhance oxygenation, and as a result, the lung is a key initial target in the S100A8/A9–NETosis loop (78, 81).

#### 2.3.2 Subacute phase: hepatic and renal dysfunction

Microvascular thrombosis and metabolic dysfunction in the liver and kidney occur in subacute sepsis, and the S100A8/A9–NETosis loop plays a significant role in these processes. The deposition of S100A8/A9 in the liver also activates Kupffer cells and hepatic stellate cells, which facilitate inflammatory and profibrotic reactions (82). Research indicates that S100A9 deficiency prevents liver damage by regulating the mitochondrial function via AKT–AMPK interactions (83). Similarly, in sepsis-induced AKI, the S100A8/A9–NETosis loop contributes to immunothrombosis within the glomerular and peritubular microvasculature, worsening reactive nitrogen species (RNS) production (32). Paquinimod, as a pharmacological intervention, has been shown to improve renal dysfunction by means of the restoration of mitochondrial dynamics and alleviating the effects of microvascular ischemia (84).

#### 2.3.3 Chronic phase: cardiac and neuroimmune dysfunction

The heart and brain are indicative of the chronic inflammation and dysfunction of the organs due to the long-term effects of continuous S100A8/A9–NETosis signaling. S100A9 triggers the NLRP3 inflammasome by the NOX–ROS and TLR4–NF-κB signaling pathways in septic cardiomyopathy, leading to metabolic dysregulation and contractile dysfunction (85, 86). Long-term exposure to S100A8/A9 in the brain causes the blood-brain barrier (BBB) to be permeable, enabling NETs and their inflammatory products to enter the central nervous system (CNS), accompanied by microglial activation, loss of synapses, and impaired cognition (87). Most mechanistic insights were created using experimental models of systemic inflammation. Experiments in sepsis-associated encephalopathy (SAE) and traumatic brain injury (TBI) models have shown that BBB integrity and neurological outcomes are improved by PAD4 knockout or pharmacological inhibition of S100A8/A9, which leads to the consideration that the loop is valuable in sepsis-related neuroimmune dysfunction (87, 88).

In general, since S100A8/A9 drives NETosis to influence the progression of sepsis, the combined detection of both can assess organ-specific functional changes better than a single marker. The above studies provide a robust basic and translational motivation for pharmacological interventions targeting the S100A8/A9 NETosis feedback loop.

### 2.4 Therapeutic targeting: strategies and challenges

#### 2.4.1 Targeting NETosis

Accumulating evidence indicates that targeting NETosis or its upstream regulator, S100A8/A9, is likely to be an effective approach to reducing excessive inflammation in sepsis (13, 30). The existing anti-NETosis strategies may be summarized into three directions: inhibiting NET formation, clearing its cytotoxic products, and encouraging degradation of NET (**Table 1**).

**Table 1:** Therapeutic strategies targeting the S100A8/A9–NETosis loop and related signaling pathways.

Target	Strategy	Mechanism of action	Representative agents	References
Targeting NETosis	Inhibiting NET formation	Inhibiting NOX	DPI, GSK2795039	(89, 123)
		Scavenging ROS	TPNs, quercetin, NAC	(71, 90, 124)
		Eliminating mtROS	mitoTEMPO, Nano AC	(91, 92)
		PAD4 inhibition or knockout	GSK484, Cl-amidine	(43, 93–95)
	Neutralizing NET-derived cytotoxic components	NE inhibition	sivelestat sodium, AAT	(97–100)
		Blocking histone citrullination	anti-CitH3 antibody	(103, 104)
	Promoting NET degradation	Nuclease-mediated DNA degradation	DNase I, PRX-119, long-acting nanoparticle DNase-1	(79, 107, 125)
Targeting S100A8/A9	Receptor blockade	Inhibiting TLR4 signaling	TAK-242 (Resatorvid)	(12, 32, 126)
		Inhibiting RAGE signaling	FPS-ZM1	(23, 126)
	Small-molecule inhibitors	Direct inhibition of S100A8/A9	paquinimod, ABR-238901	(37, 111)
		S100A8/A9 inhibitor-loaded nanoparticles	SiH/ABR@PLGA nanocomposite	(113)
	Genetic approaches	Gene knockout or deficiency	S100A8/A9 knockdown	(53)
		Gene silencing (siRNA-mediated)	S100A9-siRNA nanoparticles (MMM/RNANPs)	(112)
Dual targeting of S100A8/A9 and NETosis	Blocking the S100A8/A9–CitH3 signaling axis	Inhibiting CitH3 and S100A8/A9	hCitH3-mAb + paquinimod	(116)
	Blocking the S100A8/A9–ROS pathway	Combined inhibition of S100A8/A9 and ROS scavenging	SiH/ABR@PLGA nanocomposite	(113)

As an illustration, NOX inhibition by diphenyleneiodonium (DPI) and GSK2795039 inhibits the production of ROS and NETosis in addition to thrombosis in FcγRIIa^+^/hPF4^+^ transgenic mice (89). Correspondingly, nanoparticles based on tea polyphenols (TPNs) have a strong ability to eliminate reactive oxygen and nitrogen species, which inhibits GSDMD-mediated pyroptosis and subsequently increases the survival of septic mouse models (90). The use of mitoTEMPO clears mtROS and practically overcomes their cytotoxic effects, reducing systemic inflammation and organ damage (91). Nano acacetin (Nano AC) has been shown to preserve intestinal barrier integrity in septic rats by maintaining mitochondrial function and inhibiting NLRP3 inflammasome‑mediated pyroptosis via upregulation of thioredoxin-1 (TRX1) (92). Pharmacological inhibition of PAD4 with GSK484 or Cl-amidine significantly suppresses CitH3 and pulmonary edema in both CLP- and LPS-induced sepsis models, indicating that PAD4 is a key regulator in NET formation (43, 93–95). However, most of the existing data come from *in vivo* experiments and mouse models, and these findings have not yet been validated in clinical research. For instance, most PAD4 inhibitors are experimental compounds and lack sufficient pharmacokinetic data (96).

Apart from the NET formation, another treatment option is to neutralize the NET-derived cytotoxic elements. The cytotoxic factors present in NETs, including neutrophil elastase inhibitors (e.g., sivelestat sodium, α1-antitrypsin) and anti-histone antibodies, may decrease the effects of cytotoxic factors by depleting these factors (97–99). Sivelestat sodium has been reported to increase oxygenation and decrease mortality over 28 days in patients with sepsis-related acute respiratory distress syndrome (ARDS) (97). Nevertheless, such clinical trials have mostly been conducted in small sample sizes (50–80 cases), and therapeutic efficacy seems to be dependent on administration within a short window of time (24–72 h after disease onset) (100, 101). In line with this restriction, although the infusion of moderate dose sivelestat could inhibit NET formation in murine models of ischemia-reperfusion injury (IRI), it could not dissolve pre-formed NET-DNA scaffolds (102). Therefore, these clinical data have shown the importance of administering medication in the early stages of the disease. In murine sepsis-induced acute lung injury models caused by LPS or Pseudomonas aeruginosa, intravenous injection of a humanized anti-citrullinated histone H3 monoclonal antibody (hCitH3-mAb) blocks the CitH3–TLR2–PAD2 pathway, provides an anti-cytokine effect, reduces bacterial load, and substantially improves survival (103). However, it remains to be seen whether hCitH3-mAb can provide a similar benefit to human sepsis (104).

DNase I, which is a nuclease that breaks down the DNA generated by NET, has been proven to be effective in the alleviation of NET-derived burden and survival in several sepsis models (105). Nevertheless, DNase I has a low plasma half-life, which significantly limits its clinical usefulness. Although DNase I can degrade NETs, in trials targeting ARDS caused by COVID-19 patients, nebulized inhalation of DNase I showed a certain level of safety but did not significantly improve the oxygenation index or reduce mortality (106). Meanwhile, COVID-19 has a similar pathology to sepsis; the condition is also affected by the heterogeneity of patients, such as different causes, complications, and the use of corticosteroids. To overcome this limitation, long-acting DNase I preparations, such as PEGylated DNase I (PRX-119) and nanoparticle-based DNase I, have been prepared. These formulations are effective at lowering the circulating levels of cfDNA and neutrophil activation in ARDS and SARS-CoV-2 infection models (79, 107). However, PRX-119, as a single agent, has no effect on oxygenation and survival in ARDS mouse models (79). Although long-acting DNase I continuously degrades NETs in the body, it can lead to a large release of NET-associated toxic proteins, especially NE and S100A8/A9, into the circulation, once again triggering pro-inflammatory cascade reaction of ‘reperfusion injury’ (15). Taken together, their clinical application is limited to small therapeutic windows, and combination-based approaches are required.

#### 2.4.2 Targeting S100A8/A9

To date, various therapeutic methods have been identified to target S100A8/A9 signaling, such as blockade of its receptors (TLR4 and RAGE), direct inhibition of S100A8/A9 activity, and the use of gene or nanoparticle delivery. Taken together, these interventions have been found to have strong anti-inflammatory and organ-protective effects on preclinical models of sepsis and sterile inflammation (24, 66, 108).

Pharmacological inhibition of receptors in S100A8/A9-mediated signaling is the best studied pharmacological approach. TLR4 blocker TAK-242 (Resatorvid) and RAGE inhibitor FPS-ZM1 have a significant effect on reducing NLRP3 inflammasome activation and BBB disruption caused by S100A9 in experimental models (12, 109). Blockade of the S100A8/A9–TLR4/RAGE signaling pathways can alleviate myocardial injury and lung microvascular edema (45, 81, 85). Nevertheless, even with strong protection against endothelial and parenchymal damage, TLR4 inhibitors failed to improve mortality or inflammatory markers in sepsis patients in phase III clinical trials (110). Currently, there are no approved RAGE-targeted drugs in clinical trials for sepsis. These clinical trial failures suggest that although the TLR4/RAGE pathway has theoretical value in inflammation pathology, the complexity of the clinical setting and immune network means that inhibiting a single receptor does not necessarily translate into an effective treatment.

Small-molecule compounds that directly inhibit S100A8/A9 are also an alternative. Paquinimod and its analogue ABR-238901 directly interact with S100A8/A9 and successfully disrupt its inflammatory activity, leading to significant cardioprotective and systemic anti-inflammatory effects across multiple disease models (37, 111). These agents highlight the therapeutic prospects of targeting S100A8/A9 rather than its downstream receptors. However, there are no S100A8/A9 inhibitors undergoing clinical research in sepsis.

**Figure 3:**
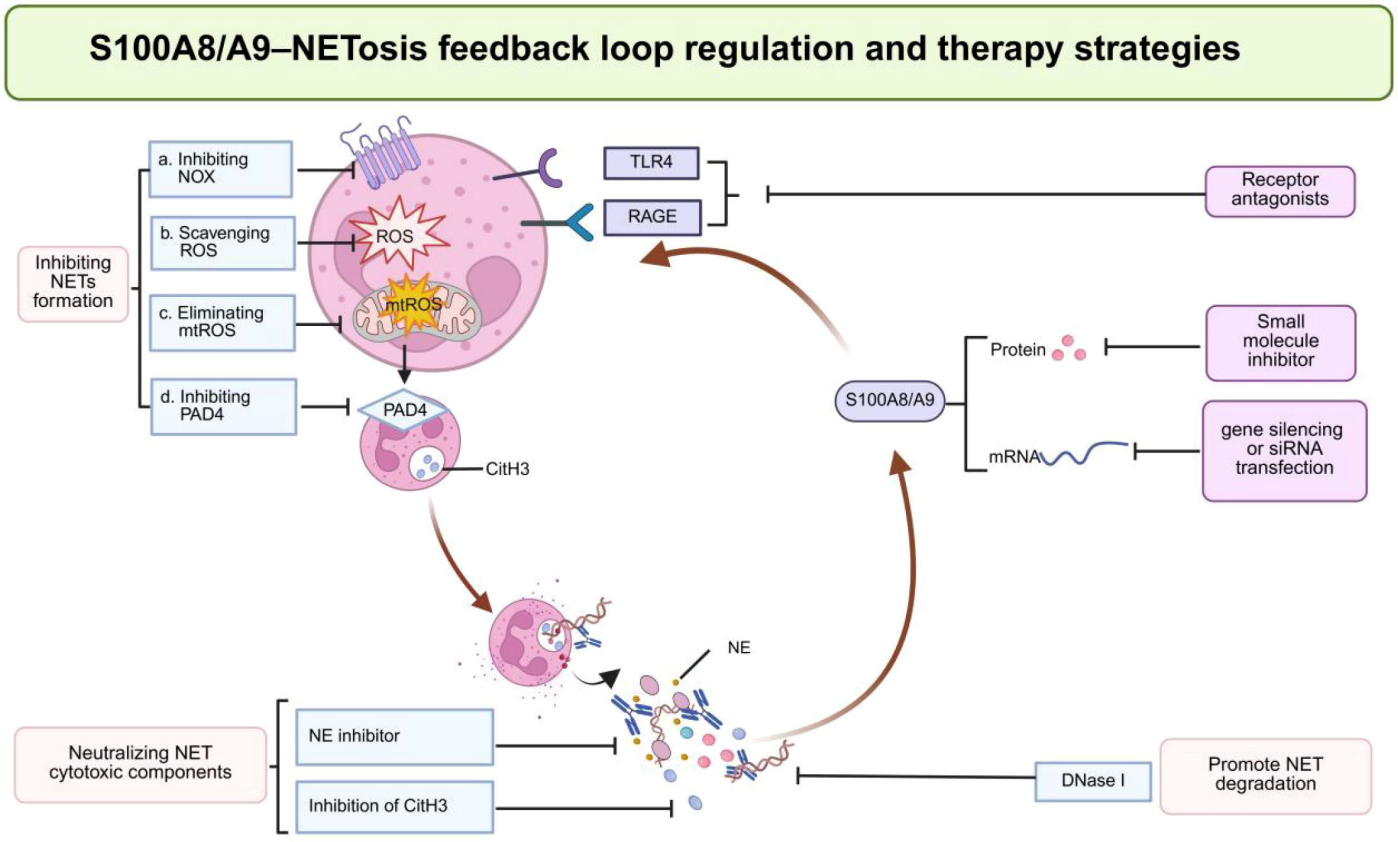
The S100A8/A9–NETosis feedback loop regulation and therapy strategies. Created using BioRender.

Additional therapeutic approaches include genetic deletion and gene interference (**Figure 3**). TLR4–NF-κB–mediated inflammatory pathways are modulated by genetic deletion of S100A9, and the NLRP3 pyroptosis signaling is suppressed; this prevents macrophage M1 polarization and reduces production of chemokines in the lungs in sepsis-induced ALI (53). More specific nanocarrier systems have also been developed to enhance the specificity of targeting and efficiency of the delivery process *in vivo*. Although intravenous delivery, hyaluronic acid– and RAGE-dual-modified, macrophage membrane-coated S100A9 siRNA nanoparticles (MMM/RNANPs) enable localized accumulation in myocardial injury tissues and potent reduction in myocardial inflammation in ischemia–reperfusion (IR) models (112). Likewise, inhibiting S100A8/A9–TLR4 signaling by intratracheal administration of ABR-2575-loaded nanocomplexes in LPS-induced sepsis-associated acute lung injury, which causes low neutrophil/macrophage inflammation, reduces the inflammatory activation pathway and produces a strong effect on the lung tissue damage (113). Moreover, antibody-based nanocarriers targeting S100A8/A9 have been shown to improve treatment outcomes in myocardial infarction and inflammatory injury models, underscoring their potential in S100A8/A9-targeted precision therapy (108, 114, 115). Nanomedicine strategies target S100A8/A9 and may improve therapeutic efficacy, safety, and personalized adaptability. However, clinical validation is still needed.

#### 2.4.3 Dual targeting of S100A8/A9 and NETosis

Although local inflammatory responses can be effectively inhibited by targeting S100A8/A9 or NETosis alone, it is difficult to disrupt the S100A8/A9–NETosis feedback loop completely. Existing evidence suggests that combined targeting of S100A8/A9 and NETosis would inhibit several elements of this loop. Moreover, the combined intervention has a strong effect. The monoclonal antibody to CitH3 (hCitH3-mAb) and the S100A8/A9 inhibitor paquinimod were used together with the experimental sepsis model, significantly inhibiting NETosis markers (MPO-DNA, CitH3, and cfDNA), which helped reduce systemic inflammation and increase survival in mice compared with monotherapy (116). Other parallel studies have also shown similar methods for treating myocardial injury. Blockage of the S100A8/A9-TLR4/RAGE-ROS-PAD4 signaling pathway has been demonstrated to have a powerful effect of reducing NETosis and release of IL-1β (85). This implies a synergistic logic of treatment. In line with this idea, a nanocomposite, which incorporates the S100A8/A9 inhibitor ABR-2575 and the reactive oxygen species scavenger SiH, showed a significant anti-inflammatory effect on septic acute lung injury (113). These results suggest that therapeutic interference with the S100A8/A9–NETosis feedback loop is a therapeutic approach to reducing cytokine storm amplification and the development of multi-organ failure in sepsis. However, extensive experimental validation remains necessary for its application in sepsis.

#### 2.4.4 Therapeutic challenges: safety, timing, and patient heterogeneity

Targeting NETosis, S100A8/A9, or both simultaneously offers promising avenues to mitigate inflammation and organ damage in preclinical models. Several challenges must be carefully considered for potential clinical translation, such as safety, timing, and patient heterogeneity.

Excessive intervention in the S100A8/A9–NETosis loop carries the risk of inducing iatrogenic immunosuppression. Thus, clinical translation must achieve a delicate balance between anti-inflammatory efficacy and immunosuppression. NETs capture pathogens through a DNA scaffold, while S100A8/A9 chelates metal ions through ‘nutritional immunity,’ together forming an important barrier in host defense (11, 18). A study using S100A8/A9-deficient mice that NETs lacking S100A8/A9 lose their fungicidal activity against *Candida albicans* (18). It underscores the risk that complete blockade may render the host susceptible to opportunistic secondary infections. Moreover, trials of nebulized DNase I treatment for COVID-19 lack significant efficacy (106), partly because it cannot neutralize already released cytotoxic components and affect the bactericidal capacity of NETosis. Therefore, an excessive targeted feedback loop can easily convert local defense mechanisms into a systemic pathological burden.

Meanwhile, interventions targeting the S100A8/A9–NETosis loop must be implemented within an optimal therapeutic window to avoid falling into the dilemma of ‘immune suppression’. This has been demonstrated in studies of TLR4 antagonists (such as Eritoran). Although they can effectively block the S100A8/A9–TLR4 signaling, they did not reduce mortality in the ACCESS trial (110). *Post hoc* analysis revealed that many patients were already in an immunosuppressed state at the time of treatment, and blocking TLR4 signaling at that point actually weakened the host’s ability to recognize secondary infections. The clinical failure of sivelestat sodium is also a typical warning: although early studies showed it could improve respiratory function, large-scale trials in patients with established acute lung injury showed no benefit (101). This suggests that inhibiting neutrophils at the late stage when injury is already established not only fails to reverse inflammation but may also impair the host’s basic defense functions. Therefore, therapies targeting the S100A8/A9–NETosis circuit should not follow a static dosing regimen. Future translational research must rely on real-time monitoring of biomarkers to ensure precise intervention at the onset of the circuit, avoiding inadvertent harm during immune exhaustion.

Furthermore, the successive failures of previous immunotherapy trials in sepsis have profoundly highlighted the core challenge of patient heterogeneity in clinical translation. Not all patients exhibit a high inflammatory state, but rather directly present a low-inflammatory phenotype characterized by immunosuppression (8, 117). Research by Scicluna et al. using blood genomic endotype indicates that sepsis is not a single disease, but rather a collection of endotypes with distinctly different molecular characteristics (118). Clinical interventions should focus on the Mars2 (hyper-inflammatory) subtype, which is characterized by excessive innate immune activation. In these patients, the surge of S100A8/A9 acts as an initiating factor, driving massive NETosis in neutrophils, thereby inducing even more S100A8/A9. Plasma levels of S100A8/A9, MPO-DNA complexes, and CitH3 can serve as biomarkers to accurately identify patients of the Mars2 subtype who are in the active phase of this loop. In contrast, for the Mars1 (immunosuppressed) subtype, which manifests as severe immunosuppression, patients usually exhibit downregulated monocyte mHLA-DR expression (119, 120). These patients are in a state of functional deficiency or hyporesponsiveness, and inhibiting this loop may exacerbate immune paralysis.

In clinical applicability, we should use point-of-care testing (POCT) to measure biomarkers for patient stratification, such as immunoturbidimetry and dry chemistry to detect plasma S100A8/A9, MPO-DNA, etc. Stratification of sepsis patients based on dominant plasma biomarkers may help identify high-risk phenotypes susceptible to immunosuppression, enabling more precise therapeutic intervention. For example, patients can be categorized into three endotypes—high S100A8/A9, high NETosis, or mixed/dual high—thereby guiding treatment options with drugs targeting S100A8/A9, NETosis inhibitors, or combination therapies. Besides, we must also combine with multiple parameters like C-reactive protein, procalcitonin, lactic acid, and the neutrophil/lymphocyte ratio (NLR) (121). NLR usually reflects the dual pathological characteristics of enhanced systemic inflammatory response and immunosuppressive state (122). But we need to combine it with other biomarkers to construct a more comprehensive assessment. Recent research using single-cell RNA sequencing (scRNA-seq) showed that monocytes expressing HLA-DR^low^ S100A9^high^ inhibit T cells in late-stage sepsis, while adding the S100A9 antagonist paquinimod can reverse immune paralysis (49). HLA-DR^low^ S100A9^high^ can serve as a marker for patient stratification, but flow cytometry is not convenient for routine use. These methods may enable rapid identification of specific subgroups truly driven by the S100A8/A9–NETosis loop.

Overall, although these therapeutic strategies show promise in preclinical models, their clinical translation faces significant challenges related to patient heterogeneity, timing of intervention, and the potential to impair host defense capabilities. Lessons can be drawn from the experiences of past failures in sepsis immunotherapy.

## 3 Conclusions and future perspectives

### 3.1 Conclusion

The discovery of the S100A8/A9–NETosis feedback loop has deepened our understanding of sepsis pathogenesis, shifting attention from linear inflammatory cascades to a self-amplifying regulatory hub. This hub combines innate immune activation, endothelial dysfunction, and multiple organ dysfunction. S100A8/A9 and NETosis-driven interactions between neutrophils, monocytes/macrophages, DCs, MDSCs, and platelets initiate immune crosstalk, thereby influencing the progression of the disease from early hyperinflammation to subsequent immune paralysis. It also contributes to the time- and location-dependent dynamics of organ injury (lungs, liver, kidneys, heart, and brain) during the progression of sepsis.

### 3.2 Knowledge gaps and future perspectives

This review integrates the novel conceptual framework of the S100A8/A9–NETosis feedback loop and proposes it as a core mechanism driving the progression of sepsis. However, despite these mechanistic and therapeutic advances, translating these findings into clinical success remains a formidable challenge. To bridge this mechanistic and translational gap, future research must address several critical knowledge gaps.

Firstly, the mechanisms of the S100A8/A9–RAGE and S100A8/A9–NOX pathways in sepsis remain unclear. Future studies can explore whether S100A8/A9 engages RAGE to drive NOX-mediated ROS production in sepsis. It is necessary to establish a septic model, using RAGE and NOX inhibitors or gene-specific knockouts, to determine the relationship between S100A8/A9 and these pathways. In addition, the contribution of the S100A8/A9–NETosis loop in other key immune subsets, such as DCs and MDSCs, remains a major knowledge gap, hindering our understanding of the broader immune landscape during sepsis progression. Future studies are needed to elucidate how this loop drives sepsis progression in these cells, which may reveal new targets for therapeutic intervention. Moreover, how this loop evolves over time, and whether its dynamic changes depend on the underlying cause (such as bacterial sepsis versus viral sepsis) or the patient’s immune status (such as hyperinflammatory state versus immunoparalysis) is unknown. It is urgent to stratify sepsis patients using biomarkers. Future clinical development should stratify patients based on combined biomarkers (such as S100A8/A9 and MPO-DNA) to obtain precise targeted treatment strategies and timing of intervention (12, 104). However, experimental detection of S100A8/A9 and MPO-DNA is mainly performed using enzyme-linked immunosorbent assay (ELISA), highlighting the need for more convenient and rapid approaches. To translate the S100A8/A9–NETosis loop from a mechanistic concept into a clinically actionable strategy for sepsis, it is crucial to address these knowledge gaps.

Finally, although the strong mechanistic foundation has been established in preclinical models, therapeutic strategies are particularly challenging. Targeting accuracy and delivery efficiency are the core challenges. Traditional systemic administration often struggles to achieve effective concentrations at the site of inflammation. Future research should focus on developing targeted delivery systems based on nanotechnology or extracellular vesicles (EVs) to achieve lesion-specific release and reduce effects on non-target tissues (127, 128). Meanwhile, the pharmacokinetics, optimal dosage, and safety of S100A8/A9 or NET-targeted interventions in humans have not yet been determined. Therefore, it is crucial to develop selective receptor antagonists and selective immunomodulators, thereby inhibiting the pathogen-induced amplification of inflammation without impairing host defense.

In conclusion, the S100A8/A9–NETosis loop has demonstrated substantial potential in the field of critical care medicine. Additionally, simultaneous targeting of S100A8/A9 and NETosis may effectively break this feedback loop, attenuating hyperinflammatory dysregulation and organ injury in sepsis. Combining mechanistic research with biomarker-based patient stratification and targeted therapy may be the key to improving and personalizing the treatment of sepsis.

## References

[1] Meyer NJ Prescott HC . Sepsis and septic shock. N Engl J Med. (2024) 391:2133–46. doi: 10.1056/NEJMra2403213. PMID: 39774315

[2] Martin-Loeches I Singer M Leone M . Sepsis: key insights, future directions, and immediate goals. A review and expert opinion. Intensive Care Med. (2024) 50:2043–9. doi: 10.1007/s00134-024-07694-z. PMID: 39531053

[3] Huang M Cai S Su J . The pathogenesis of sepsis and potential therapeutic targets. Int J Mol Sci. (2019) 20:5376. doi: 10.3390/ijms20215376. PMID: 31671729 PMC6862039

[4] Tindal EW Armstead BE Monaghan SF Heffernan DS Ayala A . Emerging therapeutic targets for sepsis. Expert Opin Ther Targets. (2021) 25:175–89. doi: 10.1080/14728222.2021.1897107. PMID: 33641552 PMC8122062

[5] Li A Ling L Qin H Arabi YM Myatra SN Egi M . Epidemiology, management, and outcomes of sepsis in ICUs among countries of differing national wealth across Asia. Am J Respir Crit Care Med. (2022) 206:1107–16. doi: 10.1164/rccm.202112-2743OC. PMID: 35763381

[6] Hotchkiss RS Monneret G Payen D . Sepsis-induced immunosuppression: from cellular dysfunctions to immunotherapy. Nat Rev Immunol. (2013) 13:862–74. doi: 10.1038/nri3552. PMID: 24232462 PMC4077177

[7] Denning NL Aziz M Gurien SD Wang P . DAMPs and NETs in sepsis. Front Immunol. (2019) 10:2536. doi: 10.3389/fimmu.2019.02536. PMID: 31736963 PMC6831555

[8] Liu D Huang SY Sun JH Zhang HC Cai QL Gao C . Sepsis-induced immunosuppression: mechanisms, diagnosis and current treatment options. Mil Med Res. (2022) 9:56. doi: 10.1186/s40779-022-00422-y. PMID: 36209190 PMC9547753

[9] Schiopu A Cotoi OS . S100A8 and S100A9: DAMPs at the crossroads between innate immunity, traditional risk factors, and cardiovascular disease. Mediators Inflammation. (2013) 2013:828354. doi: 10.1155/2013/828354. PMID: 24453429 PMC3881579

[10] Yang J Anholts J Kolbe U Stegehuis-Kamp JA Claas FHJ Eikmans M . Calcium-binding proteins S100A8 and S100A9: investigation of their immune regulatory effect in myeloid cells. Int J Mol Sci. (2018) 19:1833. doi: 10.3390/ijms19071833. PMID: 29933628 PMC6073713

[11] Wang Q Long G Luo H Zhu X Han Y Shang Y . S100A8/A9: An emerging player in sepsis and sepsis-induced organ injury. BioMed Pharmacother. (2023) 168:115674. doi: 10.1016/j.biopha.2023.115674. PMID: 37812889

[12] Su M Chen C Li S Li M Zeng Z Zhang Y . Gasdermin D-dependent platelet pyroptosis exacerbates NET formation and inflammation in severe sepsis. Nat Cardiovasc Res. (2022) 1:732–47. doi: 10.1038/s44161-022-00108-7. PMID: 35967457 PMC9362711

[13] Sprenkeler EGG Zandstra J van Kleef ND Goetschalckx I Verstegen B Aarts CEM . S100A8/A9 is a marker for the release of neutrophil extracellular traps and induces neutrophil activation. Cells. (2022) 11:236. doi: 10.3390/cells11020236. PMID: 35053354 PMC8773660

[14] Hidalgo A Libby P Soehnlein O Aramburu IV Papayannopoulos V Silvestre-Roig C . Neutrophil extracellular traps: from physiology to pathology. Cardiovasc Res. (2022) 118:2737–53. doi: 10.1093/cvr/cvab329. PMID: 34648022 PMC9586562

[15] Pignataro G Gemma S Petrucci M Barone F Piccioni A Franceschi F . Unraveling NETs in sepsis: From cellular mechanisms to clinical relevance. Int J Mol Sci. (2025) 26:7464. doi: 10.3390/ijms26157464. PMID: 40806591 PMC12346910

[16] Wang H Kim SJ Lei Y Wang S Wang H Huang H . Neutrophil extracellular traps in homeostasis and disease. Signal Transduct Target Ther. (2024) 9:235. doi: 10.1038/s41392-024-01933-x. PMID: 39300084 PMC11415080

[17] Vogl T Tenbrock K Ludwig S Leukert N Ehrhardt C van Zoelen MA . Mrp8 and Mrp14 are endogenous activators of Toll-like receptor 4, promoting lethal, endotoxin-induced shock. Nat Med. (2007) 13:1042–9. doi: 10.1038/nm1638. PMID: 17767165

[18] Urban CF Ermert D Schmid M Abu-Abed U Goosmann C Nacken W . Neutrophil extracellular traps contain calprotectin, a cytosolic protein complex involved in host defense against Candida albicans. PloS Pathog. (2009) 5:e1000639. doi: 10.1371/journal.ppat.1000639. PMID: 19876394 PMC2763347

[19] Wang S Song R Wang Z Jing Z Wang S Ma J . S100A8/A9 in inflammation. Front Immunol. (2018) 9:1298. doi: 10.3389/fimmu.2018.01298. PMID: 29942307 PMC6004386

[20] Ma M Jiang W Zhou R . DAMPs and DAMP-sensing receptors in inflammation and diseases. Immunity. (2024) 57:752–71. doi: 10.1016/j.immuni.2024.03.002. PMID: 38599169

[21] Kovačić M Mitrović-Ajtić O Beleslin-Čokić B Djikić D Subotički T Diklić M . TLR4 and RAGE conversely mediate pro-inflammatory S100A8/9-mediated inhibition of proliferation-linked signaling in myeloproliferative neoplasms. Cell Oncol (Dordr). (2018) 41:541–53. doi: 10.1007/s13402-018-0392-6. PMID: 29946821 PMC12995227

[22] Kim HJ Kim H Lee JH Hwangbo C . Toll-like receptor 4 (TLR4): new insight immune and aging. Immun Ageing. (2023) 20:67. doi: 10.1186/s12979-023-00383-3. PMID: 38001481 PMC10668412

[23] Singh H Agrawal DK . Therapeutic potential of targeting the HMGB1/RAGE axis in inflammatory diseases. Molecules. (2022) 27:7311. doi: 10.3390/molecules27217311. PMID: 36364135 PMC9658169

[24] Guan X Zha L Zhu X Rao X Huang X Xiong Y . Mechanism of action and therapeutic potential of S100A8/A9 in neuroinflammation and cognitive impairment: From molecular target to clinical application (Review). Int J Mol Med. (2025) 56:147. doi: 10.3892/ijmm.2025.5588. PMID: 40682852 PMC12289131

[25] Li T Sun H Li Y Su L Jiang J Liu Y . Downregulation of macrophage migration inhibitory factor attenuates NLRP3 inflammasome mediated pyroptosis in sepsis-induced AKI. Cell Death Discov. (2022) 8:61. doi: 10.1038/s41420-022-00859-z. PMID: 35165294 PMC8844278

[26] Wang J Wu W Wen T Zheng G Qiu G Qian H . Extracellular vesicle-bound S100A8/A9 is differentially expressed in septic shock and prompts acute lung injury. Respir Res. (2025) 26:107. doi: 10.1186/s12931-025-03181-1. PMID: 40102943 PMC11921512

[27] Modesti L Danese A Angela Maria Vitto V Ramaccini D Aguiari G Gafà R . Mitochondrial Ca^2+^ signaling in health, disease and therapy. Cells. (2021) 10:1317. doi: 10.3390/cells10061317. PMID: 34070562 PMC8230075

[28] Zheng Y Gao Y Zhu W Bai XG Qi J . Advances in molecular agents targeting toll-like receptor 4 signaling pathways for potential treatment of sepsis. Eur J Med Chem. (2024) 268:116300. doi: 10.1016/j.ejmech.2024.116300. PMID: 38452729

[29] Alves-Figueiredo H Silva-Platas C Estrada M Oropeza-Almazán Y Ramos-González M Bernal-Ramírez J . Mitochondrial Ca^2+^ uniporter-dependent energetic dysfunction drives hypertrophy in heart failure. JACC Basic Transl Sci. (2024) 9:496–518. doi: 10.1016/j.jacbts.2024.01.007. PMID: 38680963 PMC11055214

[30] Cahilog Z Zhao H Wu L Alam A Eguchi S Weng H . The role of neutrophil NETosis in organ injury: novel inflammatory cell death mechanisms. Inflammation. (2020) 43:2021–32. doi: 10.1007/s10753-020-01294-x. PMID: 32830308 PMC7443373

[31] Kuang L Wu Y Shu J Yang J Zhou H Huang X . Pyroptotic macrophage-derived microvesicles accelerate formation of neutrophil extracellular traps via GSDMD-N-expressing mitochondrial transfer during sepsis. Int J Biol Sci. (2024) 20:733–50. doi: 10.7150/ijbs.87646. PMID: 38169726 PMC10758106

[32] Tan X Zheng X Huang Z Lin J Xie C Lin Y . Involvement of S100A8/A9-TLR4-NLRP3 inflammasome pathway in contrast-induced acute kidney injury. Cell Physiol Biochem. (2017) 43:209–22. doi: 10.1159/000480340. PMID: 28854431

[33] Ansari MY Novak K Haqqi TM . ERK1/2-mediated activation of DRP1 regulates mitochondrial dynamics and apoptosis in chondrocytes. Osteoarthritis Cartilage. (2022) 30:315–28. doi: 10.1016/j.joca.2021.11.003. PMID: 34767958 PMC8792336

[34] Kerkhoff C Nacken W Benedyk M Dagher MC Sopalla C Doussiere J . The arachidonic acid-binding protein S100A8/A9 promotes NADPH oxidase activation by interaction with p67phox and Rac-2. FASEB J. (2005) 19:467–9. doi: 10.1096/fj.04-2377fje. PMID: 15642721

[35] Berthier S Nguyen MV Baillet A Hograindleur MA Paclet MH Polack B . Molecular interface of S100A8 with cytochrome b558 and NADPH oxidase activation. PloS One. (2012) 7:e40277. doi: 10.1371/journal.pone.0040277. PMID: 22808130 PMC3393751

[36] Tan C Aziz M Wang P . The vitals of NETs. J Leukoc Biol. (2021) 110:797–808. doi: 10.1002/jlb.3ru0620-375r. PMID: 33378572 PMC9059135

[37] Vlad ML Mares RG Jakobsson G Manea SA Lazar AG Preda MB . Therapeutic S100A8/A9 inhibition reduces NADPH oxidase expression, reactive oxygen species production and NLRP3 inflammasome priming in the ischemic myocardium. Eur J Pharmacol. (2025) 996:177575. doi: 10.1016/j.ejphar.2025.177575. PMID: 40180274

[38] Sun Y Xu H Gao W Deng J Song X Li J . S100a8/A9 proteins: critical regulators of inflammation in cardiovascular diseases. Front Cardiovasc Med. (2024) 11:1394137. doi: 10.3389/fcvm.2024.1394137. PMID: 39175627 PMC11338807

[39] Ryckman C McColl SR Vandal K de Médicis R Lussier A Poubelle PE . Role of S100A8 and S100A9 in neutrophil recruitment in response to monosodium urate monohydrate crystals in the air-pouch model of acute gouty arthritis. Arthritis Rheum. (2003) 48:2310–20. doi: 10.1002/art.11079. PMID: 12905486

[40] Ryckman C Gilbert C de Médicis R Lussier A Vandal K Tessier PA . Monosodium urate monohydrate crystals induce the release of the proinflammatory protein S100A8/A9 from neutrophils. J Leukoc Biol. (2004) 76:433–40. doi: 10.1189/jlb.0603294. PMID: 15107458

[41] Ding Z Du F Averitt VR Jakobsson G Rönnow CF Rahman M . Targeting S100A9 reduces neutrophil recruitment, inflammation and lung damage in abdominal sepsis. Int J Mol Sci. (2021) 22:12923. doi: 10.3390/ijms222312923. PMID: 34884728 PMC8658007

[42] Nicolai L Pekayvaz K Massberg S . Platelets: Orchestrators of immunity in host defense and beyond. Immunity. (2024) 57:957–72. doi: 10.1016/j.immuni.2024.04.008. PMID: 38749398

[43] Liu X Arfman T Wichapong K Reutelingsperger CPM Voorberg J Nicolaes GAF . PAD4 takes charge during neutrophil activation: Impact of PAD4 mediated NET formation on immune-mediated disease. J Thromb Haemost. (2021) 19:1607–17. doi: 10.1111/jth.15313. PMID: 33773016 PMC8360066

[44] Vorobjeva N Galkin I Pletjushkina O Golyshev S Zinovkin R Prikhodko A . Mitochondrial permeability transition pore is involved in oxidative burst and NETosis of human neutrophils. Biochim Biophys Acta Mol Basis Dis. (2020) 1866:165664. doi: 10.1016/j.bbadis.2020.165664. PMID: 31926265

[45] Kuipers MT Vogl T Aslami H Jongsma G van den Berg E Vlaar AP . High levels of S100A8/A9 proteins aggravate ventilator-induced lung injury via TLR4 signaling. PloS One. (2013) 8:e68694. doi: 10.1371/journal.pone.0068694. PMID: 23874727 PMC3715539

[46] Ji X Nie C Yao Y Ma Y Huang H Hao C . S100A8/9 modulates perturbation and glycolysis of macrophages in allergic asthma mice. PeerJ. (2024) 12:e17106. doi: 10.7717/peerj.17106. PMID: 38646478 PMC11032659

[47] Wang Q Shen H Wang J Wang G Zhang Y . Myeloid-specific S100A8/A9 deficiency attenuates atrial fibrillation through prevention of TLR4/NF-kB-mediated immune cell recruitment and inflammation. Front Immunol. (2025) 16:1623486. doi: 10.3389/fimmu.2025.1623486. PMID: 40977740 PMC12443547

[48] Cremers NAJ van den Bosch MHJ van Dalen S Di Ceglie I Ascone G van de Loo F . S100A8/A9 increases the mobilization of pro-inflammatory Ly6C(high) monocytes to the synovium during experimental osteoarthritis. Arthritis Res Ther. (2017) 19:217. doi: 10.1186/s13075-017-1426-6. PMID: 28969686 PMC5623958

[49] Yao RQ Zhao PY Li ZX Liu YY Zheng LY Duan Y . Single-cell transcriptome profiling of sepsis identifies HLA-DR(low)S100A(high) monocytes with immunosuppressive function. Mil Med Res. (2023) 10:27. doi: 10.1186/s40779-023-00462-y. PMID: 37337301 PMC10278311

[50] Liao YL Zhou XY Ji MH Qiu LC Chen XH Gong CS . S100A9 upregulation contributes to learning and memory impairments by promoting microglia M1 polarization in sepsis survivor mice. Inflammation. (2021) 44:307–20. doi: 10.1007/s10753-020-01334-6. PMID: 32918665

[51] van Kooten NJT Blom AB Teunissen van Manen IJ Theeuwes WF Roth J Gorris MAJ . S100A8/A9 drives monocytes towards M2-like macrophage differentiation and associates with M2-like macrophages in osteoarthritic synovium. Rheumatol (Oxford). (2025) 64:332–43. doi: 10.1093/rheumatology/keae020. PMID: 38216750 PMC11701306

[52] Franz S Ertel A Engel KM Simon JC Saalbach A . Overexpression of S100A9 in obesity impairs macrophage differentiation via TLR4-NFkB-signaling worsening inflammation and wound healing. Theranostics. (2022) 12:1659–82. doi: 10.7150/thno.67174. PMID: 35198063 PMC8825590

[53] Gong C Ma J Deng Y Liu Q Zhan Z Gan H . S100A9(-/-) alleviates LPS-induced acute lung injury by regulating M1 macrophage polarization and inhibiting pyroptosis via the TLR4/MyD88/NFκB signaling axis. BioMed Pharmacother. (2024) 172:116233. doi: 10.1016/j.biopha.2024.116233. PMID: 38308971

[54] Nagareddy PR Sreejit G Abo-Aly M Jaggers RM Chelvarajan L Johnson J . NETosis is required for S100A8/A9-induced granulopoiesis after myocardial infarction. Arterioscler Thromb Vasc Biol. (2020) 40:2805–7. doi: 10.1161/atvbaha.120.314807. PMID: 32878477 PMC7578110

[55] Monteith AJ Miller JM Maxwell CN Chazin WJ Skaar EP . Neutrophil extracellular traps enhance macrophage killing of bacterial pathogens. Sci Adv. (2021) 7:eabj2101. doi: 10.1126/sciadv.abj2101. PMID: 34516771 PMC8442908

[56] Fan X Liu Z Jin H Yan J Liang HP . Alterations of dendritic cells in sepsis: featured role in immunoparalysis. BioMed Res Int. (2015) 2015:903720. doi: 10.1155/2015/903720. PMID: 25821827 PMC4363672

[57] Lu J Sun K Yang H Fan D Huang H Hong Y . Sepsis inflammation impairs the generation of functional dendritic cells by targeting their progenitors. Front Immunol. (2021) 12:732612. doi: 10.3389/fimmu.2021.732612. PMID: 34566996 PMC8458800

[58] Li G Chen H Liu L Xiao P Xie Y Geng X . Role of interleukin-17 in acute pancreatitis. Front Immunol. (2021) 12:674803. doi: 10.3389/fimmu.2021.674803. PMID: 34594321 PMC8476864

[59] Wei Y Sun M Zhang X Zhang C Yang C Nian H . S100A8/A9 promotes dendritic cell-mediated Th17 cell response in Sjögren's dry eye disease by regulating the Acod1/STAT3 pathway. Invest Ophthalmol Vis Sci. (2025) 66:35. doi: 10.1167/iovs.66.1.35. PMID: 39808117 PMC11737457

[60] Feng WQ Zhang YC Xu ZQ Yu SY Huo JT Tuersun A . IL-17A-mediated mitochondrial dysfunction induces pyroptosis in colorectal cancer cells and promotes CD8^+^ T-cell tumour infiltration. J Transl Med. (2023) 21:335. doi: 10.1186/s12967-023-04187-3. PMID: 37211606 PMC10200054

[61] Venet F Demaret J Gossez M Monneret G . Myeloid cells in sepsis-acquired immunodeficiency. Ann N Y Acad Sci. (2021) 1499:3–17. doi: 10.1111/nyas.14333. PMID: 32202669

[62] Zheng LY Duan Y He PY Wu MY Wei ST Du XH . Dysregulated dendritic cells in sepsis: functional impairment and regulated cell death. Cell Mol Biol Lett. (2024) 29:81. doi: 10.1186/s11658-024-00602-9. PMID: 38816685 PMC11140885

[63] Fontaine M Planel S Peronnet E Turrel-Davin F Piriou V Pachot A . S100A8/A9 mRNA induction in an ex vivo model of endotoxin tolerance: roles of IL-10 and IFNγ. PloS One. (2014) 9:e100909. doi: 10.1371/journal.pone.0100909. PMID: 24956170 PMC4067416

[64] Chen D Jin Z Chu H Wu Y Bian Y Yuan T . DNASE1L3-expressing dendritic cells promote CD8^+^ T cell function and anti-PD-(L)1 therapy efficacy by degrading neutrophil extracellular traps. Cancer Cell. (2025) 43:1758–1775.e8. doi: 10.1016/j.ccell.2025.07.014. PMID: 40816293

[65] Sayyadioskoie SR Schwacha MG . Myeloid-derived suppressor cells (MDSCs) and the immunoinflammatory response to injury (Mini Review). Shock. (2021) 56:658–66. doi: 10.1097/shk.0000000000001795. PMID: 33882515

[66] Dai J Kumbhare A Youssef D McCall CE El Gazzar M . Intracellular S100A9 promotes myeloid-derived suppressor cells during late sepsis. Front Immunol. (2017) 8:1565. doi: 10.3389/fimmu.2017.01565. PMID: 29204146 PMC5698275

[67] Ostrand-Rosenberg S Huecksteadt T Sanders K . The receptor for advanced glycation endproducts (RAGE) and its ligands S100A8/A9 and high mobility group box protein 1 (HMGB1) are key regulators of myeloid-derived suppressor cells. Cancers. (2023) 15:1026. doi: 10.3390/cancers15041026. PMID: 36831371 PMC9954573

[68] Zhang W Fang X Gao C Song C He Y Zhou T . MDSCs in sepsis-induced immunosuppression and its potential therapeutic targets. Cytokine Growth Factor Rev. (2023) 69:90–103. doi: 10.1016/j.cytogfr.2022.07.007. PMID: 35927154

[69] Huang M Wu R Chen L Peng Q Li S Zhang Y . S100A9 regulates MDSCs-mediated immune suppression via the RAGE and TLR4 signaling pathways in colorectal carcinoma. Front Immunol. (2019) 10:2243. doi: 10.3389/fimmu.2019.02243. PMID: 31620141 PMC6759487

[70] von Wulffen M Luehrmann V Robeck S Russo A Fischer-Riepe L van den Bosch M . S100A8/A9-alarmin promotes local myeloid-derived suppressor cell activation restricting severe autoimmune arthritis. Cell Rep. (2023) 42:113006. doi: 10.1016/j.celrep.2023.113006. PMID: 37610870

[71] Choi SM Lee PH An MH Yun-Gi L Park S Baek AR . N-acetylcysteine decreases lung inflammation and fibrosis by modulating ROS and Nrf2 in mice model exposed to particulate matter. Immunopharmacol Immunotoxicol. (2022) 44:832–7. doi: 10.1080/08923973.2022.2086138. PMID: 35657279

[72] Yang Y Zhang X Jing L Xiao Y Gao Y Hu Y . MDSC-derived S100A8/9 contributes to lupus pathogenesis by promoting TLR7-mediated activation of macrophages and dendritic cells. Cell Mol Life Sci. (2024) 81:110. doi: 10.1007/s00018-024-05155-w. PMID: 38429401 PMC10907481

[73] Joshi A Schmidt LE Burnap SA Lu R Chan MV Armstrong PC . Neutrophil-derived protein S100A8/A9 alters the platelet proteome in acute myocardial infarction and is associated with changes in platelet reactivity. Arterioscler Thromb Vasc Biol. (2022) 42:49–62. doi: 10.1161/atvbaha.121.317113. PMID: 34809447 PMC8691374

[74] Revenstorff J Ludwig N Hilger A Mersmann S Lehmann M Grenzheuser JC . Role of S100A8/A9 in platelet-neutrophil complex formation during acute inflammation. Cells. (2022) 11:3944. doi: 10.3390/cells11233944. PMID: 36497202 PMC9738100

[75] Tang WH Hwa J . A feedback loop between platelets and NETs amplifies inflammation in severe sepsis. Nat Cardiovasc Res. (2022) 1:698–9. doi: 10.1038/s44161-022-00110-z. PMID: 35967458 PMC9362555

[76] Colicchia M Schrottmaier WC Perrella G Reyat JS Begum J Slater A . S100A8/A9 drives the formation of procoagulant platelets through GPIbα. Blood. (2022) 140:2626–43. doi: 10.1182/blood.2021014966. PMID: 36026606 PMC10653093

[77] Giles JA Greenhalgh AD Denes A Nieswandt B Coutts G McColl BW . Neutrophil infiltration to the brain is platelet-dependent, and is reversed by blockade of platelet GPIbα. Immunology. (2018) 154:322–8. doi: 10.1111/imm.12892. PMID: 29325217 PMC5979746

[78] Hiroshima Y Hsu K Tedla N Wong SW Chow S Kawaguchi N . S100A8/A9 and S100A9 reduce acute lung injury. Immunol Cell Biol. (2017) 95:461–72. doi: 10.1038/icb.2017.2. PMID: 28074060 PMC5454315

[79] Sapoznikov A Evgy Y Fux L Ruderfer I Nataf Y Hayon Y . NET degradation attenuates ricin-induced acute lung injury and protects mice from ARDS. Mol Med. (2025) 31:304. doi: 10.1186/s10020-025-01370-8. PMID: 41023796 PMC12481763

[80] Chakraborty D Zenker S Rossaint J Hölscher A Pohlen M Zarbock A . Alarmin S100A8 activates alveolar epithelial cells in the context of acute lung injury in a TLR4-dependent manner. Front Immunol. (2017) 8:1493. doi: 10.3389/fimmu.2017.01493. PMID: 29180999 PMC5693860

[81] Yu J Zhao B Pi Q Zhou G Cheng Z Qu C . Deficiency of S100A8/A9 attenuates pulmonary microvascular leakage in septic mice. Respir Res. (2023) 24:288. doi: 10.1186/s12931-023-02594-0. PMID: 37978525 PMC10655323

[82] Hou C Wang D Zhao M Ballar P Zhang X Mei Q . MANF brakes TLR4 signaling by competitively binding S100A8 with S100A9 to regulate macrophage phenotypes in hepatic fibrosis. Acta Pharm Sin B. (2023) 13:4234–52. doi: 10.1016/j.apsb.2023.07.027. PMID: 37799387 PMC10547964

[83] Zhang Y Wu F Teng F Guo S Li H . Deficiency of S100A9 alleviates sepsis-induced acute liver injury through regulating AKT-AMPK-dependent mitochondrial energy metabolism. Int J Mol Sci. (2023) 24:2112. doi: 10.3390/ijms24032112. PMID: 36768433 PMC9916677

[84] Shi W Wan TT Li HH Guo SB . Blockage of S100A8/A9 ameliorates septic nephropathy in mice. Front Pharmacol. (2023) 14:1172356. doi: 10.3389/fphar.2023.1172356. PMID: 37547329 PMC10398385

[85] Sreejit G Abdel Latif A Murphy AJ Nagareddy PR . Emerging roles of neutrophil-borne S100A8/A9 in cardiovascular inflammation. Pharmacol Res. (2020) 161:105212. doi: 10.1016/j.phrs.2020.105212. PMID: 32991974 PMC7755830

[86] Zeng M Niu Y Huang J Deng L . Advances in neutrophil extracellular traps and ferroptosis in sepsis-induced cardiomyopathy. Front Immunol. (2025) 16:1590313. doi: 10.3389/fimmu.2025.1590313. PMID: 40356926 PMC12066755

[87] Yue J Mo L Zeng G Ma P Zhang X Peng Y . Inhibition of neutrophil extracellular traps alleviates blood-brain barrier disruption and cognitive dysfunction via Wnt3/β-catenin/TCF4 signaling in sepsis-associated encephalopathy. J Neuroinflamm. (2025) 22:87. doi: 10.1186/s12974-025-03395-6. PMID: 40102948 PMC11917101

[88] Shi G Cao Y Xu J Chen B Zhang X Zhu Y . Inhibition of S100A8/A9 ameliorates neuroinflammation by blocking NET formation following traumatic brain injury. Redox Biol. (2025) 81:103532. doi: 10.1016/j.redox.2025.103532. PMID: 39929053 PMC11849670

[89] Leung HHL Perdomo J Ahmadi Z Yan F McKenzie SE Chong BH . Inhibition of NADPH oxidase blocks NETosis and reduces thrombosis in heparin-induced thrombocytopenia. Blood Adv. (2021) 5:5439–51. doi: 10.1182/bloodadvances.2020003093. PMID: 34478504 PMC9153028

[90] Chen Y Luo R Li J Wang S Ding J Zhao K . Correction to intrinsic radical species scavenging activities of tea polyphenols nanoparticles block pyroptosis in endotoxin-induced sepsis. ACS Nano. (2022) 16:4973. doi: 10.1021/acsnano.2c01759. PMID: 35238560

[91] Arulkumaran N Pollen SJ Tidswell R Gaupp C Peters VBM Stanzani G . Selective mitochondrial antioxidant MitoTEMPO reduces renal dysfunction and systemic inflammation in experimental sepsis in rats. Br J Anaesthesia. (2021) 127:577–86. doi: 10.1016/j.bja.2021.05.036. PMID: 34332740

[92] Guo NK Si LN Li PQ Gan GF . Nano acacetin mitigates intestinal mucosal injury in sepsis rats by protecting mitochondrial function and regulating TRX1 to inhibit the NLRP3 pyroptosis pathway. Int J Nanomedicine. (2024) 19:14125–41. doi: 10.2147/ijn.S497081. PMID: 39759963 PMC11699839

[93] Su X Li L Dai J Xiao B Jin Z Liu B . GSK484, a PAD4 inhibitor, improves endothelial dysfunction in mice with sepsis-induced lung injury by inhibiting H3Cit expression. Nan Fang Yi Ke Da Xue Xue Bao. (2024) 44:2396–403. doi: 10.12122/j.issn.1673-4254.2024.12.16. PMID: 39725629 PMC11683355

[94] Biron BM Chung CS O'Brien XM Chen Y Reichner JS Ayala A . Cl-amidine prevents histone 3 citrullination and neutrophil extracellular trap formation, and improves survival in a murine sepsis model. J Innate Immun. (2017) 9:22–32. doi: 10.1159/000448808. PMID: 27622642 PMC5219946

[95] Liang Y Pan B Alam HB Deng Q Wang Y Chen E . Inhibition of peptidylarginine deiminase alleviates LPS-induced pulmonary dysfunction and improves survival in a mouse model of lethal endotoxemia. Eur J Pharmacol. (2018) 833:432–40. doi: 10.1016/j.ejphar.2018.07.005. PMID: 29981294 PMC6195118

[96] Li Y Gao C Zhao J Zhao Z Xie B Zuo H . Screening of peptidyl arginine deiminase 4 inhibitors in traditional herbal medicines. Fitoterapia. (2024) 177:106095. doi: 10.1016/j.fitote.2024.106095. PMID: 38942299

[97] Wu T Wang T Jiang J Tang Y Zhang L Jiang Z . Effect of neutrophil elastase inhibitor (Sivelestat sodium) on oxygenation in patients with sepsis-induced acute respiratory distress syndrome. J Inflammation Res. (2025) 18:4449–58. doi: 10.2147/jir.S506549. PMID: 40166593 PMC11956702

[98] Pan B Li Y Liu Y Wang W Huang G Ouyang Y . Circulating CitH3 is a reliable diagnostic and prognostic biomarker of septic patients in acute pancreatitis. Front Immunol. (2021) 12:766391. doi: 10.3389/fimmu.2021.766391. PMID: 34868018 PMC8637845

[99] Cai M Deng J Wu S Cao Y Chen H Tang H . Alpha-1 antitrypsin targeted neutrophil elastase protects against sepsis-induced inflammation and coagulation in mice via inhibiting neutrophil extracellular trap formation. Life Sci. (2024) 353:122923. doi: 10.1016/j.lfs.2024.122923. PMID: 39032690

[100] Ma S Li C Gao Z Xie J Qiu H Yang Y . Effects of intravenous sivelestat sodium on prevention of acute respiratory distress syndrome in patients with sepsis: study protocol for a double-blind multicentre randomised controlled trial. BMJ Open. (2023) 13:e074756. doi: 10.1136/bmjopen-2023-074756. PMID: 37709320 PMC10503371

[101] Zhou Y Chen G Xu J Wang H Ji Q Liu A . Therapeutic effect and mechanism of sivelestat sodium on acute lung injury: a randomized controlled trial. Medicine. (2025) 104:e42703. doi: 10.1097/md.0000000000042703. PMID: 41029079 PMC13593247

[102] Kiwit A Lu Y Lenz M Knopf J Mohr C Ledermann Y . The dual role of neutrophil extracellular traps (NETs) in sepsis and ischemia-reperfusion injury: comparative analysis across murine models. Int J Mol Sci. (2024) 25:3787. doi: 10.3390/ijms25073787. PMID: 38612596 PMC11011604

[103] Ouyang W Chen Y Tan T Song Y Dong T Yu X . A citrullinated histone H3 monoclonal antibody for immune modulation in sepsis. Nat Commun. (2025) 16:7435. doi: 10.1038/s41467-025-62788-6. PMID: 40796783 PMC12343807

[104] Tian Y Li P Wu Z Deng Q Pan B Stringer KA . Citrullinated histone H3 mediates sepsis-induced lung injury through activating caspase-1 dependent inflammasome pathway. Front Immunol. (2021) 12:761345. doi: 10.3389/fimmu.2021.761345. PMID: 34950139 PMC8688857

[105] Zhang T Liu P Shen W Li C Zhao Z Wu Y . DNase I-mediated chemotactic nanoparticles for NETs targeting and microenvironment remodeling treatment of acute ischemic stroke. Adv Sci (Weinh). (2025) 12:e03689. doi: 10.1002/advs.202503689. PMID: 40536328 PMC12442667

[106] Åkesson P Mellhammar L Rasmussen M Inghammar M Jesperson S Månsson F . Aerosolized Dornase Alfa (DNase I) for the treatment of severe respiratory failure in COVID-19: a randomized controlled trial. Open Forum Infect Dis. (2025) 12:ofaf246. doi: 10.1093/ofid/ofaf246. PMID: 40365079 PMC12069806

[107] Lee YY Park HH Park W Kim H Jang JG Hong KS . Long-acting nanoparticulate DNase-1 for effective suppression of SARS-CoV-2-mediated neutrophil activities and cytokine storm. Biomaterials. (2021) 267:120389. doi: 10.1016/j.biomaterials.2020.120389. PMID: 33130319 PMC7583619

[108] Frangogiannis NG . S100A8/A9 as a therapeutic target in myocardial infarction: cellular mechanisms, molecular interactions, and translational challenges. Eur Heart J. (2019) 40:2724–6. doi: 10.1093/eurheartj/ehz524. PMID: 31334753 PMC6703152

[109] Jin H Li Z Tan S Xiao Q Li Q Ye J . Neutrophil mobilization triggers microglial functional change to exacerbate cerebral ischemia-reperfusion injury. Adv Sci (Weinh). (2025) 12:e03722. doi: 10.1002/advs.202503722. PMID: 40557450 PMC12462934

[110] Opal SM Laterre PF Francois B LaRosa SP Angus DC Mira JP . Effect of eritoran, an antagonist of MD2-TLR4, on mortality in patients with severe sepsis: the ACCESS randomized trial. Jama. (2013) 309:1154–62. doi: 10.1001/jama.2013.2194. PMID: 23512062

[111] Jakobsson G Papareddy P Andersson H Mulholland M Bhongir R Ljungcrantz I . Therapeutic S100A8/A9 blockade inhibits myocardial and systemic inflammation and mitigates sepsis-induced myocardial dysfunction. Crit Care. (2023) 27:374. doi: 10.1186/s13054-023-04652-x. PMID: 37773186 PMC10540409

[112] Lu H Wang J Chen Z Wang J Jiang Y Xia Z . Engineered macrophage membrane-coated S100A9-siRNA for ameliorating myocardial ischemia-reperfusion injury. Adv Sci (Weinh). (2024) 11:e2403542. doi: 10.1002/advs.202403542. PMID: 39264262 PMC11538685

[113] Su F Zhang C Zhang Q Shen Y Li S Shi J . Multifaceted immunomodulatory nanocomplexes target neutrophilic-ROS inflammation in acute lung injury. Adv Sci (Weinh). (2025) 12:e2411823. doi: 10.1002/advs.202411823. PMID: 39737874 PMC11848588

[114] Cai Z Xie Q Hu T Yao Q Zhao J Wu Q . S100A8/A9 in myocardial infarction: a promising biomarker and therapeutic target. Front Cell Dev Biol. (2020) 8:603902. doi: 10.3389/fcell.2020.603902. PMID: 33282877 PMC7688918

[115] Marinković G Koenis DS de Camp L Jablonowski R Graber N de Waard V . S100A9 links inflammation and repair in myocardial infarction. Circ Res. (2020) 127:664–76. doi: 10.1161/circresaha.120.315865. PMID: 32434457

[116] Dong T Ouyang W Yu X Zhao T Shao L Quan C . Synergistic inhibition of CitH3 and S100A8/A9: a novel therapeutic strategy for mitigating sepsis-induced inflammation and lung injury. Int J Immunopathol Pharmacol. (2025) 39:3946320251338661. doi: 10.1177/03946320251338661. PMID: 40390290 PMC12093008

[117] Xiao W Mindrinos MN Seok J Cuschieri J Cuenca AG Gao H . A genomic storm in critically injured humans. J Exp Med. (2011) 208:2581–90. doi: 10.1084/jem.20111354. PMID: 22110166 PMC3244029

[118] Scicluna BP van Vught LA Zwinderman AH Wiewel MA Davenport EE Burnham KL . Classification of patients with sepsis according to blood genomic endotype: a prospective cohort study. Lancet Respir Med. (2017) 5:816–26. doi: 10.1016/s2213-2600(17)30294-1. PMID: 28864056

[119] Bermejo-Martin JF Cavaillon JM Bouma H . Thirty years of mHLA-DR research in septic shock: lessons and caveats for clinical translation. Intensive Care Med. (2026) 52:138–41. doi: 10.1007/s00134-025-08220-5. PMID: 41273370

[120] Joshi I Carney WP Rock EP . Utility of monocyte HLA-DR and rationale for therapeutic GM-CSF in sepsis immunoparalysis. Front Immunol. (2023) 14:1130214. doi: 10.3389/fimmu.2023.1130214. PMID: 36825018 PMC9942705

[121] Zhang Y Peng W Zheng X . The prognostic value of the combined neutrophil-to-lymphocyte ratio (NLR) and neutrophil-to-platelet ratio (NPR) in sepsis. Sci Rep. (2024) 14:15075. doi: 10.1038/s41598-024-64469-8. PMID: 38956445 PMC11219835

[122] Wu H Cao T Ji T Luo Y Huang J Ma K . Predictive value of the neutrophil-to-lymphocyte ratio in the prognosis and risk of death for adult sepsis patients: a meta-analysis. Front Immunol. (2024) 15:1336456. doi: 10.3389/fimmu.2024.1336456. PMID: 38562922 PMC10982325

[123] Kim SK Rho SJ Kim SH Kim SY Song SH Yoo JY . Protective effects of diphenyleneiodonium, an NADPH oxidase inhibitor, on lipopolysaccharide-induced acute lung injury. Clin Exp Pharmacol Physiol. (2019) 46:153–62. doi: 10.1111/1440-1681.13050. PMID: 30403294

[124] Ding WC Chen J Li Q Ren Y Wang MM Zhang W . Quercetin confers protection against sepsis-related acute respiratory distress syndrome by suppressing ROS/p38 MAPK pathway. Chin J Integr Med. (2025) 31:1011–20. doi: 10.1007/s11655-025-3927-5. PMID: 40553253

[125] Wang CL Wang Y Jiang QL Zeng Y Yao QP Liu X . DNase I and sivelestat ameliorate experimental hindlimb ischemia-reperfusion injury by eliminating neutrophil extracellular traps. J Inflammation Res. (2023) 16:707–21. doi: 10.2147/jir.S396049. PMID: 36852300 PMC9961174

[126] Yan L Li Y Tan T Qi J Fang J Guo H . RAGE-TLR4 crosstalk is the key mechanism by which high glucose enhances the lipopolysaccharide-induced inflammatory response in primary bovine alveolar macrophages. Int J Mol Sci. (2023) 24:7007. doi: 10.3390/ijms24087007. PMID: 37108174 PMC10138623

[127] Vashist A Perez Alvarez G Andion Camargo V Raymond AD Arias AY Kolishetti N . Recent advances in nanogels for drug delivery and biomedical applications. Biomater Sci. (2024) 12:6006–18. doi: 10.1039/d4bm00224e. PMID: 39484856 PMC11528912

[128] Huang H Wang J Mao L Huang J Deng L . Neutrophil-targeted nanomedicine delivery systems: therapeutic applications and future perspectives in sepsis management. Nanoscale. (2025) 17:19987–20005. doi: 10.1039/d5nr01489a. PMID: 40843514

